# Otogenic Spontaneous pneumocephalus: case report

**DOI:** 10.5935/1808-8694.20130115

**Published:** 2015-10-08

**Authors:** Fabio Augusto Rabello, Eduardo Tanaka Massuda, Jose Antonio Apparecido de Oliveira, Miguel Angelo Hyppolito

**Affiliations:** a PhD, Medical Sciences (MD).; b PhD, Otorhinolaryngology (Assistant Physician, HCRP-FMRP - USP).; c Professor (Retired Professor, FMRP - USP).; d PhD, Medicine (Professor, FMRP-USP).; Medical School of Ribeirão Preto - University of São Paulo - University Hospital (FMRP - USP). FAEPA - HCRP - FMRP-USP.

**Keywords:** brain diseases, mastoid, abnormalities, mastoid, surgery

## INTRODUCTION

Pneumocephalus is the intracranial presence of air caused by trauma, tumor, radiation therapy, infection and, rarely, spontaneous reasons. The latter includes mastoid hyperpneumatization and the formation of a fistula between the mastoid and the cranial cavity^1^, ^2^. Congenital defects of the mastoid or of the tympanosquamous or tympanomastoid sutures allow air kept in by a valve mechanism to escape into the cranial cavity or the subperiosteal space^1^, ^3^.

Symptoms arise from compression of the meninges and the encephalon. Meningitis and compressive neurologic symptoms are potential risks connected to increased local aeration^4^, ^5^.

Diagnosis is performed with the aid of computed tomography (CT) scans. In the 12 cases published in the literature, headache, hemiplegia, otoliquorrhea, visual alterations, aphasia, and tinnitus have been described^1^, ^5^, ^6^.

Treatment aims to control symptoms and prevent complications. The communication between the mastoid and the cranial cavity may be closed with a flap or muscle fascia, cartilage, bone wax, or hydroxyapatite^3^.

## CASE REPORT

**Figure 1:**
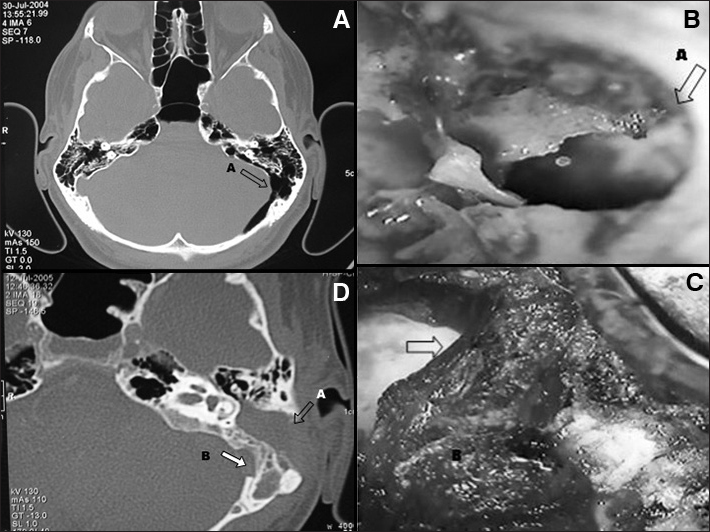
A: CT scan showing the communication between the mastoid cavity and the posterior fossa; B: Intraoperative image of a mastoidectomy showing the communication between mastoid air cells and the posterior fossa (A); C: occlusion with a temporal muscle pedicled flap; D: Six months after surgery: closure of the communication, occlusion of the mastoid and middle ear (A) and absorption of the pneumocephalus (B).

A 35-year-old male came to our center complaining of aural fullness in the left ear, nasal obstruction, and nasal pruritus persisting for years. He did not have rhinorrhea. The patient had parieto-occipital and left mastoid headaches which grew stronger when he performed the Valsalva maneuver; he also had sudden episodes of intense headache. He did not have hypacusis, autophonia, or tinnitus. The patient claimed not to have had head trauma. Physical examination did not reveal neurologic alterations. Endoscopy indicated both ears were normal. Head CT scans revealed a left extradural parietal pneumocephalus, mastoid hyperpneumatization, and extensive communication between the mastoid and the posterior cranial fossa (Figure 1A). The patient underwent subtotal mastoidectomy, obliteration of the communication between the mastoid and the posterior fossa with the placement of a flap and muscle fascia fixated with fibrin glue (Figure 1B), and occlusion of the left eustachian tube and antrum (Figure 1C).

The headaches subsided within one week, even under pressure variations or when the patient performed the Valsalva maneuver. CT scans taken six months after surgery showed complete remission of the pneumocephalus (Figure 1D).

## DISCUSSION

Only 13 cases of spontaneous pneumocephalus associated with mastoid hyperpneumatization have been described in the literature^1^, ^3^, ^4^.

Patients were aged between 20 and 78 years and the first symptom was headache after performing the Valsalva maneuver. Neurologic alterations such as aphasia, hemianopsia, otogenic cerebrospinal fluid leakage, and hemiparesis have been described in isolated cases. Auditory symptoms such as aural fullness and tinnitus were rare^5^, ^6^.

We believe this was the fourteenth case of pneumocephalus described in the literature. The patient had intermittent headaches which grew in intensity when the pressure in the eustachian tube was varied through the Valsalva maneuver; he also had aural fullness and autophonia when the headache episodes were more intense. No other symptoms were observed. Only three cases in the literature had isolated symptoms, probably related to the existence of pneumocephalus prior to the onset of neurologic symptoms and headaches^4^, ^5^, ^6^.

It has been considered whether mastoid hyperpneumatization is related to malformations of the temporal bone and headaches in patients aged between 20 and 40 years, a time in which subjects are more likely to be exposed to abrupt middle ear pressure variations and intracranial pressure increases with headaches.

Surgery to mitigate intracranial pressure has been the treatment of choice. In four of the cases described in the literature the patients were offered mastoidectomy with occlusion of the eustachian tube. Some cases require surgery through the posterior cranial fossa, conservative approaches, avoiding the Valsalva maneuver, and puncturing the pneumocephalus. No cases of recurrence have been reported when the communication between the mastoid and the cranial cavity was closed with muscle flap, muscle fascia, cartilage, bone wax, or hydroxyapatite^4^, ^5^, ^6^.

## CLOSING REMARKS

Spontaneous pneumocephalus has been associated with temporal bone defects. Mastoid hyperpneumatization with a fistula to the cranial cavity is a determining factor in the formation of pneumocephalus. Auditory symptoms are rare. Headache related to pressure variations and the Valsalva maneuver is a common and valuable finding in the diagnosis and management of neurologic manifestations and complications.
